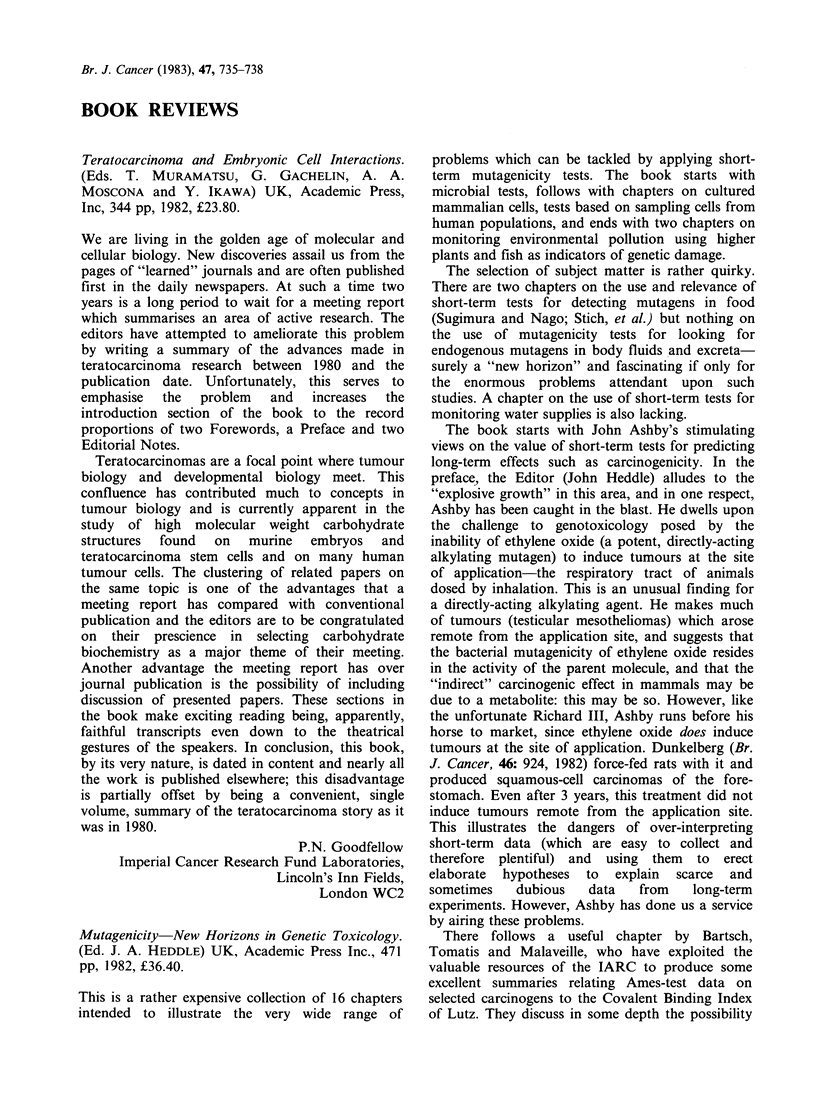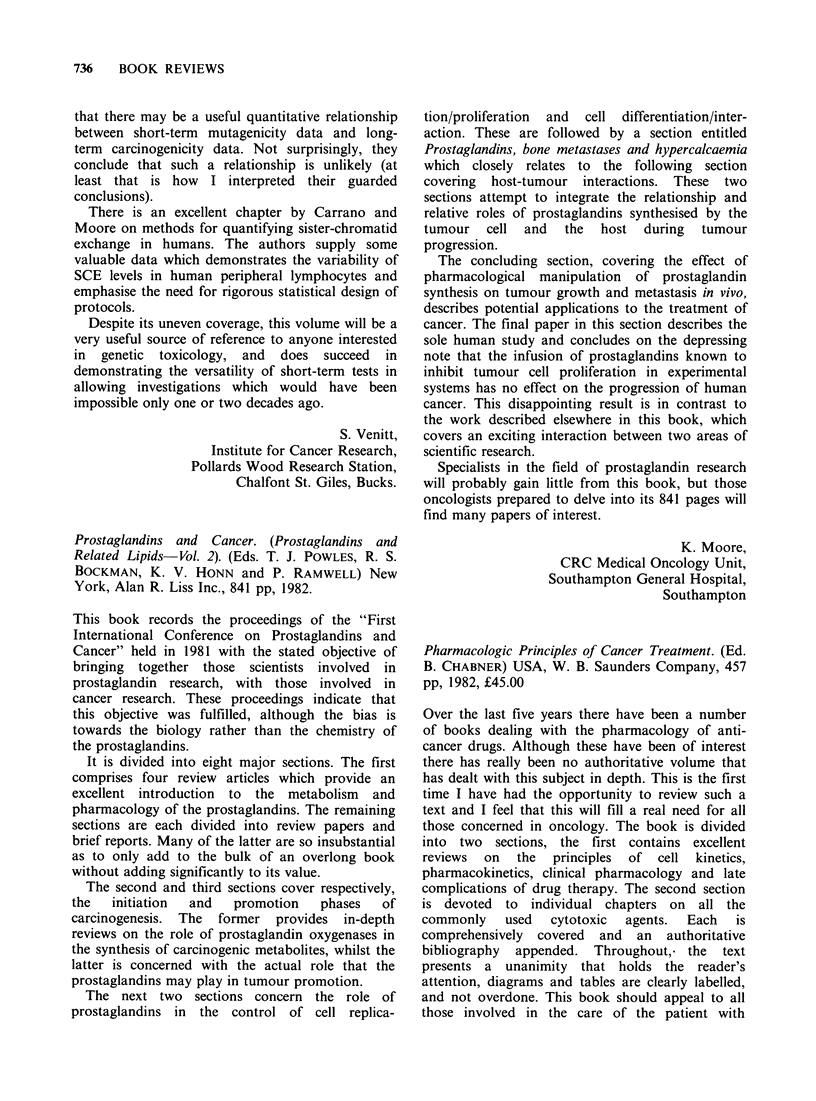# Mutagenicity—New Horizons in Genetic Toxicology

**Published:** 1983-05

**Authors:** S. Venitt


*Mutagenicity—New Horizons in Genetic Toxicology.* (Ed. J. A. HEDDLE) UK, Academic Press Inc., 471 pp, 1982, £36.40.

This is a rather expensive collection of 16 chapters intended to illustrate the very wide range of problems which can be tackled by applying short-term mutagenicity tests. The book starts with microbial tests, follows with chapters on cultured mammalian cells, tests based on sampling cells from human populations, and ends with two chapters on monitoring environmental pollution using higher plants and fish as indicators of genetic damage.

The selection of subject matter is rather quirky. There are two chapters on the use and relevance of short-term tests for detecting mutagens in food (Sugimura and Nago; Stich, *et al.)* but nothing on the use of mutagenicity tests for looking for endogenous mutagens in body fluids and excreta—surely a "new horizon" and fascinating if only for the enormous problems attendant upon such studies. A chapter on the use of short-term tests for monitoring water supplies is also lacking.

The book starts with John Ashby's stimulating views on the value of short-term tests for predicting long-term effects such as carcinogenicity. In the preface, the Editor (John Heddle) alludes to the "explosive growth" in this area, and in one respect, Ashby has been caught in the blast. He dwells upon the challenge to genotoxicology posed by the inability of ethylene oxide (a potent, directly-acting alkylating mutagen) to induce tumours at the site of application—the respiratory tract of animals dosed by inhalation. This is an unusual finding for a directly-acting alkylating agent. He makes much of tumours (testicular mesotheliomas) which arose remote from the application site, and suggests that the bacterial mutagenicity of ethylene oxide resides in the activity of the parent molecule, and that the "indirect" carcinogenic effect in mammals may be due to a metabolite: this may be so. However, like the unfortunate Richard III, Ashby runs before his horse to market, since ethylene oxide *does* induce tumours at the site of application. Dunkelberg (*Br. J. Cancer,* **46:** 924, 1982) force-fed rats with it and produced squamous-cell carcinomas of the fore-stomach. Even after 3 years, this treatment did not induce tumours remote from the application site. This illustrates the dangers of over-interpreting short-term data (which are easy to collect and therefore plentiful) and using them to erect elaborate hypotheses to explain scarce and sometimes dubious data from long-term experiments. However, Ashby has done us a service by airing these problems.

There follows a useful chapter by Bartsch, Tomatis and Malaveille, who have exploited the valuable resources of the IARC to produce some excellent summaries relating Ames-test data on selected carcinogens to the Covalent Binding Index of Lutz. They discuss in some depth the possibility that there may be a useful quantitative relationship between short-term mutagenicity data and long-term carcinogenicity data. Not surprisingly, they conclude that such a relationship is unlikely (at least that is how I interpreted their guarded conclusions).

There is an excellent chapter by Carrano and Moore on methods for quantifying sister-chromatid exchange in humans. The authors supply some valuable data which demonstrates the variability of SCE levels in human peripheral lymphocytes and emphasise the need for rigorous statistical design of protocols.

Despite its uneven coverage, this volume will be a very useful source of reference to anyone interested in genetic toxicology, and does succeed in demonstrating the versatility of short-term tests in allowing investigations which would have been impossible only one or two decades ago.

S. Venitt,
Institute for Cancer Research,
Pollards Wood Research Station,
Chalfont St. Giles, Bucks.